# Supplementary material for: Dependence in instrumental activities of daily living and its implications for older adults’ oral health
Source: PLoS One. 2021 Apr 29;16(4):e0249275. doi: 10.1371/journal.pone.0249275 (PMC8084198; doi:10.1371/journal.pone.0249275)
Supplement: Attachment — Submitted filename: Rebutal letter-02.11.2020.docx [file pone.0249275.s001.docx]

**Revision -** PONE-D-20-18747

Dear Editor,

Re: Point-by-point revision - PONE-D-20-18747

As solicitated, we have reviewed the manuscript and answered all issues raised by the reviewed. Below is a point by point explanation for all the revision performed. Attached is the revised article, with all changes marked in red, as well as, a new version of the manuscript – with not marks.

We are very pleased to have our text published at PLOS ONE. Please do not hesitate in contact us if you need any further information and or reviews.

Best regards,

Maria Vieira Lima Saintrain and co-authors

**Point by point revision -** PONE-D-20-18747

PONE-D-20-18747
Dependence in instrumental activities of daily living and its implications for older adults’ oral health
PLOS ONE

Dear Dr. Saintrain,

Thank you for submitting your manuscript to PLOS ONE. After careful consideration, we feel that it has merit but does not fully meet PLOS ONE’s publication criteria as it currently stands. Therefore, we invite you to submit a revised version of the manuscript that addresses the points raised during the review process.

Please submit your revised manuscript by Nov 05 2020 11:59PM. If you will need more time than this to complete your revisions, please reply to this message or contact the journal office at [plosone@plos.org](mailto:plosone@plos.org). When you're ready to submit your revision, log on to <https://www.editorialmanager.com/pone/> and select the 'Submissions Needing Revision' folder to locate your manuscript file.

Please include the following items when submitting your revised manuscript:

- A rebuttal letter that responds to each point raised by the academic editor and reviewer(s). You should upload this letter as a separate file labeled 'Response to Reviewers'.
- A marked-up copy of your manuscript that highlights changes made to the original version. You should upload this as a separate file labeled 'Revised Manuscript with Track Changes'.
- An unmarked version of your revised paper without tracked changes. You should upload this as a separate file labeled 'Manuscript'.

If you would like to make changes to your financial disclosure, please include your updated statement in your cover letter. Guidelines for resubmitting your figure files are available below the reviewer comments at the end of this letter.

If applicable, we recommend that you deposit your laboratory protocols in [protocols.io](http://protocols.io) to enhance the reproducibility of your results. Protocols.io assigns your protocol its own identifier (DOI) so that it can be cited independently in the future. For instructions see: <http://journals.plos.org/plosone/s/submission-guidelines#loc-laboratory-protocols>

We look forward to receiving your revised manuscript.

Kind regards,

Frédéric Denis, Ph.D.
Academic Editor
PLOS ONE

Journal Requirements:

When submitting your revision, we need you to address these additional requirements.

1. Please ensure that your manuscript meets PLOS ONE's style requirements, including those for file naming. The PLOS ONE style templates can be found at

<https://journals.plos.org/plosone/s/file?id=wjVg/PLOSOne_formatting_sample_main_body.pdf> and

<https://journals.plos.org/plosone/s/file?id=ba62/PLOSOne_formatting_sample_title_authors_affiliations.pdf>

2. Please provide additional details regarding participant consent. In the Methods section, please ensure that you have specified what type of consent you obtained (for instance, written or verbal) and whether the ethics committee approved this consent procedure. If verbal consent was obtained please state why it was not possible to obtain written consent and how verbal consent was recorded. If your study included minors, state whether you obtained consent from parents or guardians.

***More details were added to the manuscript regarding the participant consent. The text was adjusted to: “All procedures performed in studies involving human participants were in accordance with the Institutional Review Board and the national research committee and with the 1964 Helsinki declaration and its later amendments or comparable ethical standards. Written informed consent was obtained from all individual participants included in the study, which was approved by the University of Fortaleza ethics committee (Approval No. 200/2009).”***

3. Thank you for including your ethics statement:  "All procedures performed in studies involving human participants were in accordance with the Institutional Review Board (Approval No. 200/2009) and the national research committee and with the 1964 Helsinki declaration and its later amendments or comparable ethical standards. Informed consent was obtained from all individual participants included in the study.".

Please amend your current ethics statement to include the full name of the ethics committee/institutional review board(s) that approved your specific study. Please amend your current ethics statement to confirm that your named institutional review board or ethics committee specifically approved this study.

***The text was amended as suggested: “All procedures performed in studies involving human participants were in accordance with the Institutional Review Board and the national research committee and with the 1964 Helsinki declaration and its later amendments or comparable ethical standards. Written informed consent was obtained from all individual participants included in the study, which was approved by the University of Fortaleza ethics committee (Approval No. 200/2009).”***

Once you have amended this/these statement(s) in the Methods section of the manuscript, please add the same text to the “Ethics Statement” field of the submission form (via “Edit Submission”).

For additional information about PLOS ONE ethical requirements for human subjects research, please refer to <http://journals.plos.org/plosone/s/submission-guidelines#loc-human-subjects-research>.

3. Please include captions for your Supporting Information files at the end of your manuscript, and update any in-text citations to match accordingly. Please see our Supporting Information guidelines for more information: http://journals.plos.org/plosone/s/supporting-information.

***The following Caption was inserted in the end of the Manuscript.***

***S1 Appendix: STROBE Statement for the research performed—Checklist of items that should be included in reports of cross-sectional studies***

[Note: HTML markup is below. Please do not edit.]

Reviewers' comments:

Reviewer's Responses to Questions

**Comments to the Author**

1. Is the manuscript technically sound, and do the data support the conclusions?

The manuscript must describe a technically sound piece of scientific research with data that supports the conclusions. Experiments must have been conducted rigorously, with appropriate controls, replication, and sample sizes. The conclusions must be drawn appropriately based on the data presented.

Reviewer #1: Partly

Reviewer #2: Yes

2. Has the statistical analysis been performed appropriately and rigorously?

Reviewer #1: I Don't Know

Reviewer #2: I Don't Know

3. Have the authors made all data underlying the findings in their manuscript fully available?

The [PLOS Data policy](http://www.plosone.org/static/policies.action#sharing) requires authors to make all data underlying the findings described in their manuscript fully available without restriction, with rare exception (please refer to the Data Availability Statement in the manuscript PDF file). The data should be provided as part of the manuscript or its supporting information, or deposited to a public repository. For example, in addition to summary statistics, the data points behind means, medians and variance measures should be available. If there are restrictions on publicly sharing data—e.g. participant privacy or use of data from a third party—those must be specified.

Reviewer #1: Yes

Reviewer #2: No

4. Is the manuscript presented in an intelligible fashion and written in standard English?

PLOS ONE does not copyedit accepted manuscripts, so the language in submitted articles must be clear, correct, and unambiguous. Any typographical or grammatical errors should be corrected at revision, so please note any specific errors here.

Reviewer #1: Yes

Reviewer #2: Yes

5. Review Comments to the Author

Please use the space provided to explain your answers to the questions above. You may also include additional comments for the author, including concerns about dual publication, research ethics, or publication ethics. (Please upload your review as an attachment if it exceeds 20,000 characters)

Reviewer #1: Oral health is an understudied aspect of health, and additions to the scientific literature are needed. However, I identified a number of weaknesses in the manuscript that, without being addressed, substantively limit the degree to which the manuscript adds to the knowledge base.

***We appreciate your comments and suggestions. Adjustments were performed in the manuscript in order to address the issues raised by your revisions.***

p. 3, paragraph 2: The first 2 sentences don’t clearly connect to previous statements or the sense of the paragraph – they seem to be addressing different sentiments that need separate paragraphs… There are other problems with that paragraph, in terms of apparent disjointed ideas appearing. For example, the statement about associations with edentulism being followed by a statement about the prevalence rate of dependence in IADL, with no transitional connection between them.
As I read along further, a more general issue became apparent. Specifically, there is a lack of theoretical framework presented to tie together oral health and IADL. What is the mechanism that ties those factors together? The theoretical framework providing the impetus for this study must be made explicit to support the study premise.

***A new paragraph was inserted in the text, as well as a reorganization of the previous paragraph was performed in order to address the issues raised by the reviewer. Below is a transcription of the actual text.***

***“The decline in functional capacity during aging leads to difficulties in performing basic activities of daily living – ADL (bathing, dressing, feeding, transferring/walking and sphincter control) and instrumental activities of daily living – IADL (keeping track of finances, using the telephone or computer, managing medication, shopping) [1]. IADL involve more complex levels of physical functioning and neuropsychological organization than ADL [2].***

***The literature has shown an association between chronical disease and AIDL and ADL (Nunes et al., 2010), as well as between systemic disease and oral health (Kim and Amar, 2006). Periodontal disease, for instance, has been associated with cardiovascular disease, type 2 diabetes mellitus, adverse pregnancy outcomes, such as low birth weight, and osteoporosis (Kim and Amar, 2006). Thus, oral health may be indicative of systemic health, which in turn, is related to AIDL and ADL. However, the relationship between oral health and AIDL and ADL is not commonly studied, especially in the elder population. This is a relevant issue to be evaluated, as oral health should be internalized as an important health aspect of the elder. The recognition of a bidirectional relationship between oral health and AIVD, as seen between oral health and systemic health, may improve the assistance given to elders.***

***In Brazil, the rate of dependence in at least one activity of daily living (ADL) or instrumental activity of daily living (IADL) among older adults is 32.6% and the prevalence rate of exclusive dependence in IADL is 14.6% [7]. Elders can be categorized into three broad functional groups: functionally independent, frail, and functionally dependent. Although chronic illness is common among older adults, it does not necessarily lead to disability, but constitutes an important risk factor for its development [3]. Disability has a huge impact on oral health care and the treatment of functionally dependent older adults is challenging due to their inability to perform oral hygiene and their need to use multiple drugs, which create an oral environment conducive to a rapid destruction of teeth [4, 5]. Additionally, researchers have found that, in adjusted Poisson regression analysis, older age and functional disability were associated with edentulism [6].”***

This is especially needed due to the causal flow implied by your choice of outcome and independent variables. For example, on p. 10, you indicate that “…older people who used toothpaste had 5.07 times more chances of not being dependent …”. Are you really saying that the causal flow is toothpaste use ◊ dependence? It seems to me to be at least as likely (actually, more likely) that independence ◊ toothpaste use.

***We thank the reviewer for bringing up this important point. We have adjusted the manuscript in order to clarified this issue. The objective of the present study was to evaluate the existence of an association between oral health and AIVD, which we found. At this point, we cannot imply causality in this association, and the text was adjusted in this manner. As for the text in page 10, what we are saying is that use toothpaste is associated with not being dependent. The text has been modified to the following:***

***“Table 3 shows that dependence in IADL was also significantly associated with chewing and swallowing difficulties (p=0.011)^2^, voice changes (p=0.044)^2^, edentulism (p=0.011)^2^, number of teeth (p= 0.021)^2^, use of toothbrush (p<0.001)^2^, use of toothpaste (p<0.001)^2^, and visit to the dentist in the previous year (p=0.020)^2^. Other associations with oral issues were also identified. Dependent older people were 2.19 times more likely to present chewing and swallowing difficulties and 2.22 times more likely to have voice changes. Older people who used toothpaste had 5.07 times less chances of being dependent and those who did not have dental visits in the previous year were 2.42 times more likely to be dependent.”***

Specific hypotheses or, at the very least, research questions, are not presented, which, combined with the scant statistical analysis description presented, makes evaluation of statistical method appropriateness difficult, especially with regards to Table 4.

***We thank the reviewer for asking for a clear state on the research question in the manuscript. For us, it was so clear in our minds that we believed it to be clear in the article as well. As for reviewer suggestion, we have added the following text in the manuscript: “Thus, we wanted to know if oral, general health and chronicles issues were related to instrumental activities of daily living in the studied population. Furthermore, we wanted to investigate the variables that remained associated with AIVD when the associated issues were evaluated together, point out to indicators that might be use in the elder population.”***

Sample size justification was apparently predicated on contingency table analyses planned – justification is needed that the parameters used are realistic and represent clinically substantive associations.

***We were not sure about the issue raised by the reviewer regarding our sample size calculation. We utilized common and broadly used parameters for the sample size calculation for finite populations, as follows:***

***The sample size was estimated considering the number of older adults (N=242,430) in the city of Fortaleza in the year 2012 as reported in Brazil’s National Health System Database [18]. A minimum sample size was estimated considering an expected maximum proportion of 20%, a significance level of 5% (95% confidence interval), and a maximum permissible error of 5%. The following formula for finite populations was used:***

***n = ___N . k^2^ . p . q_____***
***e^2^ . (N-1) + k^2^ . p .q***
***Where: n= sample size; N= population; k= parameter of significance level; p= % of occurrence; q= difference of 1 – p; e= sampling error.***
***The formula estimated a minimum sample size of 246 older people.***
Justification is needed for the cut-points used to produce ordered categories – for example, citation for the cut-points defining independence groups; rationale for the age groupings utilized, etc.

***The cut-points for AIVD on dependente, semi-dependent and independente were based on Pinto et al. (2016) classification. This has been added to the manuscript as follows: “The maximum score is 27. Individuals with scores of 19-27 points are classified as independent. Scores of 10-18 indicate assisted independence and scores of 1-9 points indicate dependence. (Pinto et al., 2016)”.***

***As for age, we know that the age pyramid is commonly classified for every five years, however, in order to facilitate inferential analysis, the age of the elderly was categorized into the age groups of 60-69, 70-79, 80 and more, following the example of Sousa et al., 2018. [18]. It is important to know that in Brazil, elders older than 80years are considered an “extra” elder group and have especial civic rights. The manuscript was adjusted to insert the clarification for the age cut point as follows: “To facilitate inferential analysis, the age of the elderly was categorized into the age groups of 60-69, 70-79, 80 and more, following the example of Sousa et al., 2018. [18]”.***

Is there information related to the validity and reliability of the Lawton and Brody scale in a Brazilian population? How about validity and reliability of selected other measures (e.g.: general health items, oral health items)?

***According to Santos & Virtuoso Júnior (2008), who analyzed the reliability of the Scale of Instrumental Activities of Daily Living (IADL) developed by Lawton and Brody and adapted to Brazilian context. Concerning the stability of the measures, they can be classified as almost perfect agreement, both for the reliability (Ricc=0.89) as for the objectivity (Ricc=0.80). Oral health was measured utilizing the ICSB, which was created for the Brazilian community by one of the co-authors (Saintrain, 2007) of the present manuscript and has been used in a series of studies (Saintrain & Vieira, 2012; Saintrain et al., 2018a; Saintrain et al., 2018b). Other general and oral health items, as performed in several other studies, were directly asked to the study population. Validity and reliability are not commonly measure for this type of questions.***

***ICSB References:***

***Saintrain, MVL. Proposta de um indicador comunitário em saúde bucal. RBPS 2007; 20 (3):199-204 doi:10.5020/18061230.2007.p199*** [***https://doi.org/10.5020/1026***](https://doi.org/10.5020/1026)

# ***Saintrain, MVL. Vieira, APGF. Application of the Community Oral Health Indicator by Non-Dental Personnel and Its Contribution to Oral Healthcare. July 2012.*** [***PLoS ONE***](https://www.researchgate.net/journal/1932-6203_PLoS_ONE) ***7(7):e39733. DOI:*** [***10.1371/journal.pone.0039733***](https://www.researchgate.net/deref/http%3A%2F%2Fdx.doi.org%2F10.1371%2Fjournal.pone.0039733)

***Saintrain MVL, Saintrain SV, Sampaio EGM,*** [***Ferreira***](https://pubmed.ncbi.nlm.nih.gov/?term=Ferreira+BSP&cauthor_id=30009412) ***BSP,*** [***Nepomuceno***](https://pubmed.ncbi.nlm.nih.gov/?term=Nepomuceno+TC&cauthor_id=30009412) ***TC,*** [***Frota***](https://pubmed.ncbi.nlm.nih.gov/?term=Frota+MA&cauthor_id=30009412) ***MA,*** [***Vieira-Meyer***](https://pubmed.ncbi.nlm.nih.gov/?term=Vieira-Meyer+AP&cauthor_id=30009412) ***APGF***

***Older adults’ dependence in activities of daily living: Implications for oral health. Public Health Nurs. 2018;00:1–9.*** [***https://doi.org/10.1111/phn.12529***](https://doi.org/10.1111/phn.12529)

***Saintrain MVL, Bezerra TMM, Santos FS, Saintrain SV, Pequeno LL, Silva RM, Vieira-Meyer APGF. Subjective well-being and oral discomfort in older people. International Psychogeriatrics (2018), 30:10, 1509–1517 © International Psychogeriatric Association 2018 doi:10.1017/S1041610218000145***

***The following text was added to the manuscript as follows: Concerning the stability of the measures, regarding the Instrumental Activities of Daily Living (IADL) developed by Lawton and Brody and adapted to Brazilian context, almost perfect agreement has been shown, both for the reliability (Ricc=0.89) as for the objectivity (Ricc=0.80) (Santos & Virtuoso Júnior, 2008).***

I think the discussion of recommendations with regards to oral health for patients who are dependent (p. 13) may be rather simplistic. Given that such patients are dependent on others (caregivers), it seems like one explanation would be that the dependence/disability is the cause of the poor oral hygiene and oral health, and that the caregivers, might need intervention as much as the patient… That reasoning does come out on page 14.

***We fully agree with the reviewer. We have added some extra points in the text, as follows: “Furthermore, due to the common dependence level of these patients, including for selfcare, it is important to motivate and train caregivers on oral health care. Oral health issues may commonly be left, by caregivers, to a second plane of importance due the challenges brought by other general health issues.”***

I was confused about the discussion at the bottom of page 15, where you talk about a program tailored for caregivers – are you saying that the positive findings for that study support an underlying premise that poor oral health in dependent patients may be due to inadequate oral hygiene provided by caregivers? That is a logical conclusion, given that the dependent patient is dependent for care on others – just need to explain what you mean more clearly…

***We were actually trying to state that a tailored educational program focusing on caregivers have a positive impact on dependent elder’s oral health. We have adjusted the manuscript in order to clarify this issue. “The findings of the present research showed that functional dependence in IADL is associated with oral hygiene and behaviors that maintain oral health, and therefore, is an important health indicator in older adults. Oral health problems can have profound effects on general health and well-being. Furthermore, oral pain and problems with eating, chewing, smiling and communicating due to missing, discolored or damaged teeth have a major impact on functional ability and older people’s daily lives [14]. Thus, as mentioned before, investments in oral health care through caregiver’s motivation and training may have a positive impact of elder’s health. In this regard, researchers have demonstrated that an educational program tailored to caregivers had a positive impact on the oral health of dependent older adults, observed by the improved oral hygiene parameters, such as plaque index and tongue coating [33].”***

Reviewer #2: Thank you for your submission and yes I agree that we need to assess the status of older population in order to better serve their needs.
You mentioned that some of your questionnaires are validated what about the others, where they validated? If not, how can you justify their use and reliability?

***We only utilized questionnaires that were either validated or broadly used. Some of the questionnaires, despite not being validated in Brazil, have been broadly used in published research performed in the country.***

-Were your examiners calibrated? If yes, how and how is their agreement. If not, how reliable is your data?

***The examiners were properly trained by the co-author MVLS to apply the questionnaires and the ICSB, following adequate protocols for this purpose. The ICSB was created and validated by two co-authors of this manuscript (MVLS and APGFVM), which assures that the training was performed as advocated by the creators of the indicator and that the data is reliable. Furthermore, the examiners (n=?) were closely accompanied by the co-author MVLS during data collection. The following text was inserted in the manuscript “The examinations were performed under natural light with the help of a wooden spatula (tongue depressor) – examiners wore personal protective equipment. The two examiners who performed this evaluation were trained and closely accompanied by the co-author and gold standard (one of the ICSB creators) MVLS. The minimal accepted kappa values for intra and inter examiner was 0.8.”***

-Where you able to match data from supposes? How does marriage play a role in assessing dependance?

***Regarding the point raised by the reviewer, we have added some information in the discussion part of the manuscript: “A relationship was observed between marital status and AIVD independence. It is not clear the path by which marital status plays a role on dependence, however, a possibility is related to the elders’ quality of life and mental status. According to Gutiérrez-Vega et al. (2018), married older adults had the highest quality of life in social relationships when compared to other groups. They state that marital status may play an important role when analyzing quality of life among older adults, suggesting that being married may offer a protective mechanism against depressive symptoms and therefore against mental illnesses during late adulthood.”***

***Gutiérrez-Vega M, Esparza-Del Villar OA, Carrillo-Saucedo IC, Montañez-Alvarado P. The Possible Protective Effect of Marital Status in Quality of Life Among Elders in a U.S.-Mexico Border City. Community Ment Health J. 2018;54(4):480-484. doi:10.1007/s10597-017-0166-z***

-There were some instances that you used causation as a term instead of association. Causation could not be established at this stage so please correct accordingly.

***We thank the reviewer for bringing up this important point. We have adjusted the manuscript in order to clarified this issue. The objective of the present study was to evaluate the existence of an association between oral health and AIVD, which we found. At this point, we cannot imply causality in this association, and the text was adjusted in this manner.***

-While you addressed generalizability as a limitation but it was not part of your conclusion statement. Please modify your conclusion so that it is clear that it does not apply to all population.

***Thank you for calling our attention for this issue. We have amend the conclusion in order to clarify that the findings relate to elders living in the city of Fortaleza.***

***“Our findings demonstrated that functional dependence in instrumental activities of daily living can be considered an indicator of oral health status in older adults living in Fortaleza. Functional disability was associated with older age, cardiovascular diseases, speech problems, chewing and swallowing difficulties, use of medication and brushing deficiency. Therefore, health promotion and disease prevention and rehabilitation actions should be carried out at all levels of health care considering the needs of this population group in order to improve oral hygiene and oral health-friendly behaviors”.***

6. PLOS authors have the option to publish the peer review history of their article ([what does this mean?](https://journals.plos.org/plosone/s/editorial-and-peer-review-process#loc-peer-review-history)). If published, this will include your full peer review and any attached files.


If you choose “no”, your identity will remain anonymous but your review may still be made public.


**Do you want your identity to be public for this peer review?** For information about this choice, including consent withdrawal, please see our [Privacy Policy](https://www.plos.org/privacy-policy).

Reviewer #1: No

Reviewer #2: No
