# Supplementary material for: Dependence in instrumental activities of daily living and its implications for older adults’ oral health
Source: PLoS One. 2021 Apr 29;16(4):e0249275. doi: 10.1371/journal.pone.0249275 (PMC8084198; doi:10.1371/journal.pone.0249275)
Supplement: Attachment — Submitted filename: Rebutal letter - AIVD - 31.01.2021.docx [file pone.0249275.s002.docx]

**Response to Reviewers**

Dear Editor,

We are happy to know that our manuscript is being considered for publication at PLOS ONE. We read the reviewers’ comments and adjusted the manuscript. Below we bring a point-by-point response to the reviewers. Please do not hesitate in contacting us if you need any further information.

Best regards,

Maria Vieira Saintrain and Co-authors

**Point-by-point response to reviewers’ comments**

Reviewers' comments:

Reviewer's Responses to Questions

**Comments to the Author**

1. If the authors have adequately addressed your comments raised in a previous round of review and you feel that this manuscript is now acceptable for publication, you may indicate that here to bypass the “Comments to the Author” section, enter your conflict of interest statement in the “Confidential to Editor” section, and submit your "Accept" recommendation.

Reviewer #2: All comments have been addressed

Reviewer #3: All comments have been addressed

2. Is the manuscript technically sound, and do the data support the conclusions?

The manuscript must describe a technically sound piece of scientific research with data that supports the conclusions. Experiments must have been conducted rigorously, with appropriate controls, replication, and sample sizes. The conclusions must be drawn appropriately based on the data presented.

Reviewer #2: Yes

Reviewer #3: Partly

3. Has the statistical analysis been performed appropriately and rigorously?

Reviewer #2: I Don't Know

Reviewer #3: Yes

4. Have the authors made all data underlying the findings in their manuscript fully available?

The [PLOS Data policy](http://www.plosone.org/static/policies.action#sharing) requires authors to make all data underlying the findings described in their manuscript fully available without restriction, with rare exception (please refer to the Data Availability Statement in the manuscript PDF file). The data should be provided as part of the manuscript or its supporting information, or deposited to a public repository. For example, in addition to summary statistics, the data points behind means, medians and variance measures should be available. If there are restrictions on publicly sharing data—e.g. participant privacy or use of data from a third party—those must be specified.

Reviewer #2: Yes

Reviewer #3: Yes

5. Is the manuscript presented in an intelligible fashion and written in standard English?

PLOS ONE does not copyedit accepted manuscripts, so the language in submitted articles must be clear, correct, and unambiguous. Any typographical or grammatical errors should be corrected at revision, so please note any specific errors here.

Reviewer #2: Yes

Reviewer #3: No

Reviewer #3: Background section

The background summary of research leading to the study’s aim is not entirely clear as written. While the study’s aim is to explore associations between degrees of functionality among aging adults in relationship with indicators of oral health, there is a lot of additional clinical considerations that are presented but in a disjointed manner. For example, discussion of chronic disease, edentulism, periodontal disease, disability, and disability are mentioned, but the connection the team of authors is making is not presented in a logical manner.

**Thank you for your comments. We have reorganized the background section in order to clarify the points you raised. Changes are highlighted in the manuscript and transcribed below.**

***“The decline in functional capacity during aging leads to difficulties in performing basic activities of daily living – ADL (bathing, dressing, feeding, transferring/walking and sphincter control) and instrumental activities of daily living – IADL (keeping track of finances, using the telephone or computer, managing medication, shopping) [1], where IADL involve more complex levels of physical functioning and neuropsychological organization than ADL [2].***

***Disability has a huge impact on oral health care and the treatment of functionally dependent older adults is challenging due to their inability to perform oral hygiene and their need to use multiple drugs, which create an oral environment conducive to a rapid destruction of teeth [3, 4]. On the other hand, oral diseases represent a silent epidemic that disproportionately affects older people, with caries and periodontal diseases standing out as the two greatest health threats and the most common chronic diseases for the cause of tooth loss among adults [5], which in turn, has the potential to affect elders dependency, specially related with proper nutrition. For the WHO, a minimum of 20 teeth are necessary for proper oral function/nutrition [6].***

***Poor oral health in older people is mainly manifested in high rates of tooth loss, dental caries, periodontal diseases, xerostomia (dry mouth) and other conditions resulting from poor oral hygiene [7]. Oral diseases are undoubtedly a global public health problem linked to social and economic changes, and their ongoing neglect in global health policy highlights the need to address oral diseases among other non-communicable diseases (NCDs) as a global health priority [8].***

***According to the Global Burden of Disease Study 2016, oral diseases affected circa 3.58 billion people worldwide, and caries of the permanent teeth were the most prevalent of the conditions assessed. The cumulative burden of oral conditions dramatically increased between 1990 and 2015. The number of people with untreated oral conditions rose from 2.5 billion in 1990 to 3.5 billion in 2015, with a 64% increase in DALYs (disability-adjusted life year) due to oral conditions throughout the world [9]. Severe periodontal disease, which results mainly from poor oral hygiene and which can lead to tooth loss, was the 11^th^ most prevalent disease globally [10]. Researchers have found that, in adjusted Poisson regression analysis, older age and functional disability were associated with edentulism [11]. Thus, it seems that oral health effects and is affected by elders’ functionality.***

***The increase in life expectancy has attracted growing interest in the effect of oral health on general health outcomes, especially those related to functionality, well-being and mortality [12]. Oral health and general health share common risk factors related to diet, the use of tobacco, and the excessive consumption of alcohol and the solutions to control oral disease are to be found through shared approaches with integrated chronic disease prevention [13].***

***In this regard, public health solutions for oral diseases are most efficient when they are integrated with those for other NCDs and other national public health programs. The importance of oral health goals was first emphasized in 1981 by WHO as part of the program Health for All by the year 2000 [14, 15] and most recently the WHO, jointly with the FDI World Dental Federation and the International Association for Dental Research (IADR), formulated goals for oral health by the year 2020 [16].***

***Furthermore, the WHO Global Oral Health Program is aligned with the Global NCD agenda and the Shanghai Declaration on promoting health in the 2030 Agenda for Sustainable Development [17]. According to Petersen [13], the WHO Oral Health program gives priority to research helping correct the so called 10/90 gap which relates to the fact that only 10%of funding for global health research is allocated to health problems that affect 90% of the world population. The WHO Oral Health Program focuses on stimulating oral health research in the developed and developing world to reduce risk factors and the burden of oral disease, and to improve oral health systems and the effectiveness of community oral health programs. Building and strengthening research capacity in public healthcare highly recommended by WHO for effective control of disease and the socioeconomic development of any given country.***

***In Brazil, the rate of dependence in at least one ADL or IADL among older adults is 32.6% and the prevalence rate of exclusive dependence in IADL is 14.6% [18]. Population aging can be categorized into three broad functional groups: functionally independent, frail, and functionally dependent. Chronic and oral health diseases have a greater potential to impact frail and functionally dependent older people. However, it is important to understand that, although chronic illness is common among older adults, it does not necessarily lead to disability, but constitutes an important risk factor for its development [19].***

***In view of what was outlined above, there is an evident need for collaboration between the medical and dental fields and between professionals in both fields in order to make healthcare provision systems more effective [20]. Therefore, understanding the dimensions of the impact in terms of functional impairment caused by poor oral and general health is critical for the provision of adequate health care, communication and health education [21]. Given that, the objective of the present study was to assess the relationship between IADL and Oral Health of older people.”***

The statement: understanding dimensions of the negative impact of functional impairment caused by poor oral and general health is critical for providing adequate healthcare and health education. Yet, given the cross sectional study design, determination of causality is not possible. A clear presentation of how oral health has risen to be a global health priority (is this the first WHO global health NCD agenda to include oral health?) does not directly convey if the status of addressing oral health needs among older adults is only now gaining recognition and the impetus behind it.

**More information was inserted in the introduction section in order to address this issue. This can be seen below and is highlighted in the manuscript.**

***“The increase in life expectancy has attracted growing interest in the effect of oral health on general health outcomes, especially those related to functionality, well-being and mortality [12]. Oral health and general health share common risk factors related to diet, the use of tobacco, and the excessive consumption of alcohol and the solutions to control oral disease are to be found through shared approaches with integrated chronic disease prevention [13].***

***In this regard, public health solutions for oral diseases are most efficient when they are integrated with those for other NCDs and other national public health programs. The importance of oral health goals was first emphasized in 1981 by WHO as part of the program Health for All by the year 2000 [14, 15] and most recently the WHO, jointly with the FDI World Dental Federation and the International Association for Dental Research (IADR), formulated goals for oral health by the year 2020 [16].***

***Furthermore, the WHO Global Oral Health Program is aligned with the Global NCD agenda and the Shanghai Declaration on promoting health in the 2030 Agenda for Sustainable Development [17]. According to Petersen [13], the WHO Oral Health program gives priority to research helping correct the so called 10/90 gap which relates to the fact that only 10%of funding for global health research is allocated to health problems that affect 90% of the world population. The WHO Oral Health Program focuses on stimulating oral health research in the developed and developing world to reduce risk factors and the burden of oral disease, and to improve oral health systems and the effectiveness of community oral health programs. Building and strengthening research capacity in public healthcare highly recommended by WHO for effective control of disease and the socioeconomic development of any given country.”***

Are there epidemiologic studies to quantify years of life lost among individuals with periodontal disease, edentulism, medication-related xerostomia? This support would strengthen the main tenet of this research and as presented, the value of answering the research question is not entirely clear and convincing.

**Yes, there is some data on DALYs related to oral health conditions. Those were inserted in the text and are presented below:**

***“According to the Global Burden of Disease Study 2016, oral diseases affected circa 3.58 billion people worldwide, and caries of the permanent teeth were the most prevalent of the conditions assessed. The cumulative burden of oral conditions dramatically increased between 1990 and 2015. The number of people with untreated oral conditions rose from 2.5 billion in 1990 to 3.5 billion in 2015, with a 64% increase in DALYs (disability-adjusted life year) due to oral conditions throughout the world [9]. Severe periodontal disease, which results mainly from poor oral hygiene and which can lead to tooth loss, was the 11^th^ most prevalent disease globally [10]. Researchers have found that, in adjusted Poisson regression analysis, older age and functional disability were associated with edentulism [11]. Thus, it seems that oral health effects and is affected by elders’ functionality.”***

Within the Methods section, the first sentence is very convoluted:
All procedures performed in studies involving human participants were in accordance with the Institutional Review Board (Approval No. 200/2009) and the national research committee and with the 1964 Helsinki declaration and its later amendments or comparable ethical standards.
It includes 4 uses of ‘and’.

**Thank you for point this out. We adjusted the text as follows: *“All procedures performed in studies involving human participants were in accordance with the national research committee, as per approval No. 200/2009 from the University of Fortaleza Institutional Review Board. This committee follows the 1964 Helsinki declaration and its later amendments or comparable ethical standards. Informed consent was obtained from all individual participants included in the study.”***

The second paragraph in this section (p5) is a single sentence. Typically, a developed paragraph will have 3 sentences.
Again, in the sentence:
All the participants were informed of the objectives and procedures of the study and anonymity of participants and confidentiality of information were guaranteed.
The quality of writing could be improved with additional proofreading.
There is no need to include the STROBE checklist as a supplemental file nor to reference it within the abstract. It is a recognized checklist that can be mentioned within the manuscript and readers can seek details if interested.

**Thank you for your attention in all the details. We have adjusted the text as follows: *“All the participants were informed of the objectives and procedures of the study. Participants anonymity and confidentiality of information were guaranteed. Data were individually collected and analyzed using:”***

Concerning the instrumentation-
The statement:
The semi-structured questionnaire addressing sociodemographic data (age, sex, race, marital status, education and income), general health (poor, fair, very good), self-reported 6 diseases and oral discomfort data (dry mouth, difficulty in chewing and swallowing food, taste of food, burning mouth sensation, pain without apparent reason, swelling of the mouth).

1. Was a validated general health scale not considered (vs semi-structured questionnaire)?

**None of the general health scale known by the authors addressed all the information we were interested in. Thus, we opted for the semi-structured questionnaire. Nevertheless, we based the oral health questions on the WHO Oral Health Questionnaire for Adults.**

2. Was regimen of medication not assessed? As types of medications is related to xerostomia, for example.

**Unfortunately, not. We agreed with the reviewer that this could’ve added to a broader analysis and discussion of the results. Nevertheless, we believe that the amount of information gathered in our study is robust for our study objective.**

3. What about water source and fluoride exposure?

**This information was not collected. However, Fortaleza has a fluoridated public water system. Nevertheless, the control of this fluoridation in the municipality or the water ingestion habits (mineral or public water) of the studied population is unknown.**

4. Why not ask about nutritional and behavioral risks, such as use of alcohol and/or tobacco products? Soft-drink consumption? Any regional/national drinks that may affect tooth enamel (such as carbonated drinks, kombucha, etc?)

**We did ask about tobacco and alcohol consumption, which is presented in table 2. Nutritional consumption is an interesting, yet complex, to analyze and point direct causality to oral health disease.**

5. For the next statement about the ICSB-And items within the Community Oral Health Indicator (Indicador Comunitário de Saúde Bucal – ICSB) may be scientifically validated for specificity, sensitivity, but overall, within the context of overall health and functioning-how do the items correspond in terms of functioning/potential functional impairment? This validated indicator checks the number of teeth, visible dental cavities, presence of calculus, gingival inflammation, residual root, soft tissues injuries, use and need of dental prosthesis, use of toothbrush and toothpaste, and visits to the dentist [16]. The examinations were performed under natural light with the help of a wooden spatula (tongue depressor) – examiners wore personal protective equipment.
**what about flossing behavior? Use of water picks/syringes for flushing gums? Gingival bleeding? Fluoride exposure?

**According to the World Health Organization-WHO (1992), a minimum of 20 teeth are necessary for proper oral function/nutrition. A large number of missing teeth, dental decay, residual dental roots, tartar and gingival inflammation, among other oral issues, can affect the elders’ oral health and their ability to keep proper oral hygiene and their daily activities. Thus, the ICSB is able to identify issues with potential impact on elders’ AVD and AIVD.**

**Fluoride exposition through fluoridated dental paste was also investigated. However, flossing habits and the existence of gingival bleeding was not questioned.**

RESULTS section
1.The first statement:
Participants were 280 older people aged 60-96 years, with a mean age of 69.9 (SD±7.2) years.
Again, it is advisable to seek service from an external native English speaker, as the writing could be more fluid. For example, the study sample consisted of 280 older adults ranging from 60-96 years of age….

**Thank you for your suggestion. English was revised and this statement now reads *“The study sample consisted of 280 older adults ranging from 60-96 years of age, with a mean age of 69.9 (SD±7.2) years.”***

2. There is no frame of reference to indicate that your sample is reflective of the region overall-or if your study sample characteristics were skewed in any way.

**The information below was added to the text in order to clarify the sample calculation.**

***The sample size was estimated considering the number of older adults (N=242,430) in the city of Fortaleza in the year 2012 as reported in Brazil’s National Health System Database [18]. A minimum sample size was estimated considering an expected maximum proportion of 20%, a significance level of 5% (95% confidence interval), and a maximum permissible error of 5%.***

3. The unit of ‘one minimum wage’ does not make sense and is included in presentation of your results (and within Table 1). It is not clear what this means.

**Brazilian minimum wage is R$ 1,100.00 - approximately U$ 200.00. This information was added to table 1.**

4. Table 1 is missing closing parentheses within income values

**Thank you for pointing this up. We have adjusted the text.**

5. There was no previous indication that alcohol and smoking behavior were assessed, yet results concerning these exposures are presented in this section.

**We have added information on this issue on the Materials and methods section as follows: *“1. Semi-structured questionnaire addressing sociodemographic data (age, sex, race, marital status, education and income), general health (poor, fair, very good), self-reported diseases and oral discomfort data (dry mouth, difficulty in chewing and swallowing food, taste of food, burning mouth sensation, pain without apparent reason, swelling of the mouth), as well as alcohol and smoking habits.”.***

6. There is no contextual information provided in previous sections of manuscript to indicate why voice changes would be a significant indicator of oral health status and how it is assessed. Not sure why gingival bleeding would not be part of the assessment.

**The voice box (larynx) is made of cartilage, muscle and mucous membranes located at the top of the trachea and the base of the tongue. If the vocal cords become inflamed, develop growths or become paralyzed, they can't work properly, and one may develop a voice disorder. A voice change can be a sign of throat dehydration, Neurological disorders, or consequence of habits like alcohol consumption and tobacco usage. Furthermore, voice change can also be linked with oral discomfort. Gingival inflammation was assessed. We agree with the reviewer that gingival bleeding assessment could improve our study – we will definitely consider its inclusion in future studies.**

**Changes were made in the manuscript, as follows:**

***“Furthermore, the increased PR estimates between dependence in IADL and difficulty chewing and swallowing (2.19), voice changes (2.22), edentulous (2.30), use of toothbrush (4.90), use of toothpaste (5.07) and visit to the dentist in the previous year (2.42) demonstrates a close relationship between disability and oral health preventive measures. Our voice is an extremely sensitive indicator of our general health and emotional status [31]. The voice box (larynx) is made of cartilage, muscle and mucous membranes located at the top of the trachea and the base of the tongue. If the vocal cords become inflamed, develop growths or become paralyzed, they can't work properly, and one may develop a voice disorder. A voice change can be a sign of throat dehydration, Neurological disorders, or consequence of habits like alcohol consumption and tobacco usage. Furthermore, voice change can also be linked with oral discomfort. Considering that dental caries and periodontal disease are associated with lifestyle, patients should be encouraged to visit the dentist regularly in addition to being provided with dental health guidance in each visit, as health education has short-term effects [32].”***

7. I am assuming active caries (lesions)

**For the ICSB, dental caries are active caries lesions or non-restored caries cavities.**

8. For the indicator-pain with no apparent reason-is this general reported pain within the oral cavity-including palette, tongue? Does this include reported throat pain?

Pain within bones (mandible? Sinuses?)

**The question was “do you feel pain for no apparent reason”? Thus, it includes anything that the responder believes to be pain without apparent reason.**

9. The statement:
In addition, older adults with cardiovascular disorders were 1.77 more likely to be dependent in IADL than those without cardiovascular disorders and older adults who did not use toothbrush were 2.57 times more likely to be dependent in IADL than those who used toothbrush. Scientific research supports that periodontal disease is significantly associated with cardiovascular health, yet, presentation of this known relationship is not clear in the background section of paper. Also, there is a comma instead of a period for the value 1.77 included in Table 4

**Thank you for pointing that up. We have adjusted the text accordingly.**

Discussion section

1. The statement:
   The present study stands out for showing that dependence in IADL causes harms to the oral health of older adults.
   This statement cannot be made within the context of the study design. There is no ability to determine if IADL ‘causes’ any oral health manifestations as this study’s goal is to explore if significant associations between key variables exist.

**We fully agree with the reviewer. The statement was adjusted as follows: *“The present study stands out for showing a relationship between dependence in IADL and oral health of older adults”.***

2. Continuing in the first paragraph:
As in most epidemiological studies, there was a greater participation of women and a higher rate of women with dependence in IADL, but there was no significant difference between sexes.
Any citation?

**We have also adjusted the statement in order to clarify what we wanted to say. The changes are highlighted in the manuscript.**

***“Gender did not appear to have any influence on IADL dependency.”***

3. Statement:
This finding differs from a study that demonstrated that women have a greater incidence of disability in IADL than men, and that this difference is maintained even after controlling for social vulnerability among women and presence of chronic diseases [2].
It is very important to provide greater context into your study sample-and how it reflects characteristics of the general population and if there was an oversample of women?

**Changes were done in the manuscript in order to address this issue*: “The present study stands out for showing a relationship between dependence in IADL and oral health of older adults. Gender did not appear to have any influence on IADL dependency. This finding differs from a study that demonstrated that women have a greater incidence of disability in IADL than men, and that this difference is maintained even after controlling for social vulnerability among women and presence of chronic diseases [2]. According to the latest IBGE census, women represent 60,7% of the elder population in Fortaleza [26]. Thus, through authors’’ perspective, our female sample (65%) it comparable with the city reality and did not seem to influence our findings regarding IADL in the elder population.”***

4. In the next paragraph, starting with “In the present study….”, results of various studies are cited-but it is important to note if these are aging studies conducted in Brazil specifically. The structures/clinical supports of older adult populations vary greater in terms of national context and this is not presented in terms of your discussion points.

**We have modified the manuscript in order to follow the reviewers’ suggestion: “*In the present study, the most prevalent age group and the mean age are similar to those of another Brazilian study, where the mean age was 70.04 (±7.89) years and most people were aged 60-69 years [27]. The percentage of 13.2% of older people with different degrees of dependence in their IADL is similar to that reported in the literature for Brazil [28, 18]. However, a study shows that 46.3% of a population of 289 older people had moderate to severe dependence in IADL [29]. It is important to know that the characteristics identified regarding dependence in IADL suggest a complex causal relationship that points to the need for preventive actions specifically designed to address different factors and improve older people’s quality of life [27]. Nonetheless, it is important to point out that all the studies just mentioned were carried out in Brazil. As the structures, clinical supports and family care of older adult populations may vary in terms of national context and culture, it is important to have these issues in mind when extrapolating the study findings.”***

5. Be consistent with use of acronyms-IADL should be used from the first time it is introduced. In the discussion section alone-the ADL/IADL is frequently spelled out

**Thank you for pointing that out. We have revised the manuscript and adjusted it according to your suggestion.**

6. The background information regarding items included in your analyses would be very helpful if presented in the earliest section of your paper. For example:

Poor oral health in older people is mainly manifested in high rates of tooth loss, dental caries, periodontal diseases, xerostomia (dry mouth) and other conditions resulting from poor oral hygiene [30].

**Thank you for your comment. We have inserted this information in the introduction section.**

Conclusion section
1. The first sentence overstates value of study:
Our findings demonstrated that functional dependence in instrumental activities of daily living can be considered an indicator of oral health status in older adults.
While your study may yield results that associations exist; however, a more sophisticated subsequent study would be warranted to build upon and advance the cross-sectional study design utilized in this paper.

**We have adjusted the conclusion statement as suggested by the reviewer.**

***“Our findings demonstrated that functional dependence in IADL is related to oral health status in older Brazilian adults. Functional disability was associated with older age, cardiovascular diseases, speech problems, chewing and swallowing difficulties, use of medication and brushing deficiency. Therefore, health promotion and disease prevention and rehabilitation actions should be carried out at all levels of health care considering the needs of this population group in order to improve oral hygiene and oral health-friendly behaviors.”***

Given that this draft manuscript was a revise and resubmit, of which I have not previously reviewed, I do not think it is publication worthy in its current state. The language/writing does not clearly present the study parameters in a clear manner, and the value of the findings are not always accurate.

**We hope that the changes/adjustments made in the manuscript make you reconsider the above statement. We are very pleased that our manuscript is under consideration at PLOS ONE and willing to make any further adjustments if necessary.**

7. PLOS authors have the option to publish the peer review history of their article ([what does this mean?](https://journals.plos.org/plosone/s/editorial-and-peer-review-process#loc-peer-review-history)). If published, this will include your full peer review and any attached files.


If you choose “no”, your identity will remain anonymous but your review may still be made public.


**Do you want your identity to be public for this peer review?** For information about this choice, including consent withdrawal, please see our [Privacy Policy](https://www.plos.org/privacy-policy).

Reviewer #2: No

Reviewer #3: No
